# Automated identification of early to mid-stage Parkinson’s disease using deep convolutional neural networks on static facial images

**DOI:** 10.3389/fmedt.2025.1655199

**Published:** 2025-09-15

**Authors:** Ni Yang, Jing Liu, Lin Wang, Jiajun Ding, Lingzhi Sun, Xianghua Qi, Yitong Lu, Wei Yan

**Affiliations:** ^1^ Department of First Clinical Medical College, Shandong University of Traditional Chinese Medicine, Jinan, China; ^2^ College of Rehabilitation Medicine, Shandong University of Traditional Chinese Medicine, Jinan, China; ^3^ Qingdao Traditional Chinese Medicine Hospital, Qingdao Hiser Hospital Affiliated of Qingdao University, Qingdao, China; ^4^ School of Design, Shanghai Jiao Tong University, Shanghai, China; ^5^ Affiliated Hospital of Shandong University of Traditional Chinese Medicine, Jinan, China

**Keywords:** Parkinson's disease, identification, facial images, deep convolutional neural network, deep learning

## Abstract

**Objective:**

This study investigates deep convolutional neural networks (CNNs) for automated detection of early to mid-stage Parkinson's disease (PD) from static facial images, aiming to explore non-invasive, cost-effective approaches for early diagnosis and remote monitoring.

**Methods:**

2,000 facial images were collected from PD patients and healthy controls, followed by data augmentation to expand the dataset to 6,000 images. After randomly dividing the dataset into training and test sets according to 8:2, five CNN architectures were fine-tuned and assessed. Model performance was assessed by accuracy, precision, recall, specificity, F1 score, and area under the ROC and PR curve (AUC). Grad-CAM visualization techniques were applied to identify the discriminative facial regions associated with PD.

**Results:**

ResNet18 achieved the best overall performance, yielding an F1 score of 99.67% across metrics. MobileNetV3 also performed robustly, particularly excelling in recall (99.00%), suggesting its suitability for high-sensitivity screening applications. EfficientNetV2 demonstrated stable convergence and competitive classification performance (F1 score: 96.30%), while VGG16 exhibited balanced performance with rapid convergence. Inception-v4 showed relatively lower accuracy and greater variability, indicating a potential risk of overfitting. Grad-CAM heatmaps revealed that the most predictive facial regions across models were concentrated around the eyes, lips, and nose, consistent with PD-related hypomimia.

**Conclusion:**

CNNs, particularly ResNet18 and MobileNetV3, exhibit significant potential for the automated identification of PD from facial imagery. These models offer promising avenues for developing scalable, non-invasive screening tools suitable for early detection and remote healthcare delivery, providing significant clinical and social value in the context of aging populations.

## 1 Introduction

Parkinson's disease (PD), the second most prevalent neurodegenerative disorder (1), has witnessed a substantial upsurge in both prevalence and incidence rates over the past few decades (2). Projections suggest that by 2050, the global case count will exceed 25.2 million with the aging population (3), imposing a colossal strain on public health infrastructures. Despite the typical clinical manifestations of PD, early diagnosis remains a major challenge. Existing diagnostic criteria, including the UK Brain Bank criteria (4) and the MDS clinical diagnostic criteria (5), are predominantly reliant on subjective clinical evaluations. Patients are typically discovered only after the onset of overt motor symptoms. By this juncture, more than half of the dopaminergic neurons in the substantia nigra may have been depleted, thereby forfeiting the prime window for early intervention (6). Conventional biomarkers, such as cerebrospinal fluid alpha-synuclein assays and dopamine transporter imaging, can improve diagnostic accuracy but are constrained by high costs, invasiveness, and accessibility, rendering them unsuitable for large-scale screening initiatives (7). Consequently, developing objective, affordable, and accessible methods to detect markers of PD is of great clinical and public health significance for early detection and disease monitoring.

The typical symptoms of PD include a series of non-motor symptoms and motor impairments such as resting tremor, muscle rigidity, and bradykinesia. In recent years, hypomimia (8), as one of the important phenotypic features of PD, has been attracting increasing attention. Patients frequently present with a “masked face,” characterized by reduced blinking, passive lip separation, and flattened nasolabial folds (9). While these features can impair social interactions, they may also serve as potential early biomarkers for PD diagnosis (10).

With the advancement of artificial intelligence and computer vision, facial manifestations of PD are increasingly being transformed into quantifiable and structured data (11, 12). This enables the in-depth exploration of subtle facial muscle dynamics unique to PD patients (13). Convolutional neural networks (CNNs), known for their capabilities in automatic feature extraction, high representational power, robustness, and spatial feature modeling, have been extensively adopted in facial analysis tasks (14). They further benefit from techniques such as data augmentation and transfer learning, which enhance generalization performance (15). Building on these developments, our study explored the classical CNN models for early to mid-stage PD detection based on static facial images. This approach aims to provide a non-invasive, cost-effective, and scalable solution for Early large-scale screening and remote patient management of PD.

## 2 Related works

Initial efforts largely relied on facial landmark features. For example, Hou et al. (16) proposed a method combining facial geometric and texture descriptors, such as Euclidean distances, angles, Local Binary Patterns, and Gray-Level Co-occurrence Matrices, achieving 93.6% accuracy and an F1 score of 93.8% using data from 70 PD patients and 70 healthy controls (HC). Rajnoha et al. (17) employed 128-dimensional facial embedding vectors with Random Forest and XGBoost classifiers, reporting an accuracy of 67.33% on 50 PD-control pairs. Grammatikopoulou et al. (18) introduced two indices of facial expressiveness (HSI1 and HSI2) based on selfies collected via smartphones. Using keypoint detection through Google Face API and Microsoft Azure Face API, they achieved a sensitivity of 0.89 and a specificity of 0.73 for HSI2.

Several studies have adopted video-based analysis with temporal modeling. Bandini et al. (19) analyzed facial landmark trajectories in videos to quantify motion entropy. Gómez et al. (11) introduced a phase-based representation of facial expressions, utilized ResNet50 with transfer learning to extract Action Unit features, and optimized the feature space via triplet loss. This approach achieved 87.3% accuracy on video data from 30 PD patients, which was an improvement of 3.6% over baseline methods. Further advancing this trajectory, Valenzuela et al. (20) proposed an end-to-end spatiotemporal model upon a 3D CNN, analyzing facial movements during vowel pronunciation. The model attained 91.87% classification accuracy on 16 PD-control pairs.

The advent of deep learning has further improved diagnostic performance by enabling the extraction of high-dimensional semantic features. Calvo-Ariza et al. (13) combined traditional descriptors like Histogram of Oriented Gradient, and Local Binary Patterns with deep learning architectures, achieving 80.4% accuracy under pleasant expressions. Hou et al. (12) utilized a label-free 2D video model incorporating facial geometry and texture features and reported an F1 score of 88% using Random Forest and Support Vector Machine classifiers. Jin et al. (21) extracted 106 facial landmarks using Face++ in analyzing jitter-based features, and that Support Vector Machine yielded an F1 score of 99%, while Long-Short Term Memory achieved 86.76% accuracy. A study employing the Semantic Feature based Hypomimia Recognition network applied end-to-end deep learning to the Smile Video dataset, achieving an accuracy of 99.39% (22). In parallel, growing attention has been paid to privacy and computational efficiency. Jiang et al. (23) integrated Paillier homomorphic encryption with edge AIoT devices to perform secure video analysis on encrypted recordings from 52 PD patients, achieving an identification accuracy of 95% between patients treated with deep brain stimulation before and after. Due to the limited dataset, enhancing the training accuracy and generalization capacity of the model poses a significant challenge. Therefore, Huang et al. (24) used StarGAN to synthesize facial expressions of PD patients and combined these with a Swin Transformer to integrate multi-modal features. Their model achieved 100% diagnostic accuracy on data from 95 PD patients.

A summary of previous studies is presented in Table 1. Despite their contributions, several common limitations persist. Many models were developed using small sample sizes, resulting in poor generalizability. Additionally, facial manifestations in PD are closely associated with disease severity (25). In the early stages, patients may present only subtle signs that often escape detection by both family members and clinicians. In contrast, these features become markedly more pronounced in the advanced stages, by this time more than 50% of dopaminergic neurons in the substantia nigra may have been lost, missing the best opportunity for early intervention (6). Most studies did not specify the clinical stage of the PD patients included, and few have developed high-performance deep learning models capable of detecting early to mid-stage PD using simple static facial images. To address these gaps, we explored deep learning frameworks based on classical CNN models for the recognition of early and mid-stage PD based on static facial imagery, to facilitate early, large-scale auxiliary screening and diagnosis.

**Table 1:** Summary of previous literature on facial recognition for Parkinson's disease.

Approach	Year	Source of data	Model (s)	Performance	Metrics	Cite
Machine learning	2017	17 PD and 17 HC	The classifiers of CK + database and the Radboud FACES DAtabase with a 10-Fold Cross-Validation strategy	The average accuracy of 88%. and 98% for happiness, 90% for disgust, 88% for anger, 84% for neutral, and 74% for sadness	Neutral, happiness, anger, disgust, and sadness in video	(19)
	2018	50 PD and 50 HC	Random forest, XGBoost, and decision trees	The decision tree algorithm achieved the best accuracy with 67.33%	Simple static face pictures	(17)
	2019	23 PD and 11 HC	The Google Face API and Microsoft Face API	The sensitivity of 79% and a specificity of 82% for the Google, while for Microsoft the results were 89% and 73%	Smiling and right/left eye open in Selfie Photo	(18)
	2021	70 PD and 70 HC	Random forest, support vector machines, and k-nearest neighbor	The highest accuracy at 86% in the random forest model using texture features	Poker face and smiling in videos	(12)
	2021	70 PD and 70 HC	Support vectormachine	93.6% accuracy and 93.8% F1 score	Facial video (texture features)	(16)
	2022	FacePark-GITA database (11) (30 PD and 24 HC)	Support vector machine	72.8%, 75.8%, and 80.4% accuracy for anger, surprise, and happiness	happiness, surprise, and angriness in video	(13)
Deep learning	2020	33 PD and 31 HC	Long short-term memory	86.76% accuracy	smile expression in video	(21)
	2021	47 PD and 39 HC	VGG and ResNet with the semantic loss	99.39% accuracy	Facial video from neutral expressions to smiles	(22)
	2023	VGGFace2, EmotioNet, and FacePark-GITA database	ResNet50 Freeze 75 (transfer learning from face analysis to action units recognition)	87.3% accuracy	smile, anger, surprise, left eye wink, or right eye wink in video	(11)
	2023	95 PD (adopt the StarGAN to synthesize the premorbid normal facial expression images)	Models in ImageNet-1K and ImageNet-21K	100% accuracy in both EfficientNetV2-Small and EfficientNet-B7 model	The synthesized neutral, anger, disgust, fear, happiness, sadness, and surprise facial images	(24)

## 3 Methods

**Figure 1:**
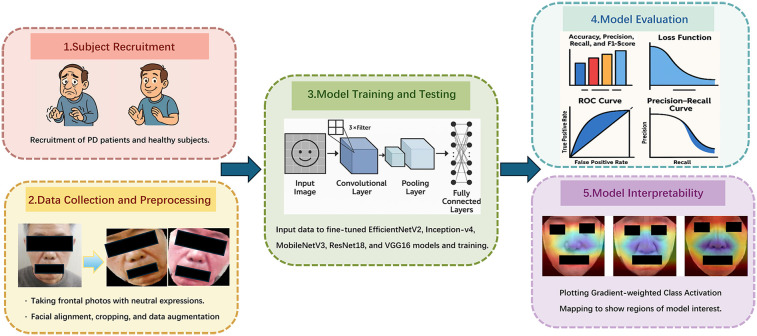
The flowchart of our research method.

Figure 1 shows the flowchart of our research method. This study has been approved by the Ethics Committee of Shandong University of Traditional Chinese Medicine Affiliated Hospital (2024-136-YJS). Our research performed in accordance with the Declaration of Helsinki. Informed consent has been obtained from all participants and/or their legal guardians, including the extra consent for the publication of identifying images.

### 3.1 Subject recruitment

#### 3.1.1 PD patients

Between March 2024 and June 2025, a total of 208 PD patients were recruited from the Department of Neurology at the Affiliated Hospital of Shandong University of Traditional Chinese Medicine. Five standardized facial images were obtained from each patient. Recruit PD patients according to the following criteria:

1. Inclusion Criteria:
  a. Patients met the diagnostic criteria for idiopathic PD established by the International Parkinson and Movement Disorder Society in 2015 (5).
  b. Disease stage ranged from 0 to 3 on the Hoehn and Yahr scale (26), indicating early to mid-stage PD.
2. Exclusion Criteria:
  a. Patients with comorbid depression, anxiety, or psychotic disorders as defined by the Diagnostic Criteria and Treatment Guidelines for Depression, Anxiety, and Psychotic Disorders in PD (27).
  b. Patients diagnosed with multiple system atrophy, progressive supranuclear palsy, or other Parkinson-plus syndromes.
  c. Patients with secondary parkinsonism due to infection, toxins, vascular lesions, drug effects, or traumatic injury.
  d. Patients with significant intracranial pathology (e.g., tumors, inflammatory lesions, history of brain surgery, extensive infarction, or cerebral hemorrhage), or with severe systemic diseases.
  e. Patients exhibiting motor fluctuations, such as the “on-off” phenomenon.
  f. Individuals of non-Asian ethnicity, those with abnormal skin pigmentation, or those presenting with facial abnormalities due to conditions such as facial paralysis.

#### 3.1.2 Healthy control subjects

During the same period, 1,013 healthy volunteers were recruited from the same hospital. One standardized facial image was collected from each participant. Recruit healthy participants according to the following criteria:

1. Inclusion Criteria:
  a. Participants were aged between 45 and 80 years, in good general health, and free from major organic diseases or psychiatric disorders.
  b. No history of persistent discomfort or significant decline in functional capacity over the past three months; able to maintain normal daily life and work activities.
2. Exclusion Criteria:
  a. Presence of any abnormal clinical findings or suspected illnesses that required exclusion.
  b. History of long-term regular medication use, blood donation within the past three months, surgical procedures within the past month, or excessive intake of alcohol, tea, coffee, or other caffeinated beverages.
  c. Individuals of non-Asian descent.
  d. Subjects with facial scarring or deformities due to trauma or other conditions.

### 3.2 Data collection

Facial images were acquired using a dual-camera system (Huawei, Beijing, China) equipped with 20-megapixel and 16-megapixel sensors. The raw images were captured at a 2K resolution of 1,860 × 2,160 pixels. Standardized clinical procedures were followed: Participants were seated indoors under natural lighting, without hats or masks, with long hair tied back to ensure full facial exposure. They were instructed to maintain a neutral expression and gaze directly forward. Additional demographic and clinical information—such as region of origin, age, education level, and treatment history—was recorded for each participant.

### 3.3 Dataset preprocessing

All preprocessing and analyses were performed using Python scripts developed and executed within the PyCharm environment (https://www.jetbrains.com/pycharm/). As the data were collected from clinical settings, preprocessing steps were essential to prepare the images for deep learning applications.

Initially, raw images underwent cleaning, cropping, and data augmentation. Facial regions were extracted from each image based on 68 facial landmarks, ensuring that only the complete face (excluding the background) was retained. To improve model robustness and generalization, two augmented images were generated from each original image using a combination of the following random techniques:

a. Horizontal flipping, to simulate left-right symmetry.
b. Vertical flipping, to increase dataset variability.
c. Random rotation within a range of −30° to +30°, enhancing rotation invariance.
d. Random adjustment of brightness, contrast, sharpness, saturation, and hue, to mimic different lighting environments and camera settings.
e. Random cropping followed by resizing to a fixed resolution, improving scale and perspective invariance.

Finally, all images were resized and normalized to a consistent shape of 256 × 256 × 3 pixels.

### 3.4 Model training and testing

We applied the PyTorch deep learning framework (https://www.pytorch.org) to develop five CNNs for the classification of PD: EfficientNetV2, Inception-v4, MobileNetV3, ResNet18, and VGG16.

EfficientNetV2 (28) begins with a standard 3 × 3 convolutional layer (stride = 2), followed by multiple stacked MBConv modules from stages two through eight. The final stage includes a 1 × 1 convolution, an average pooling layer, and a fully connected layer. This architecture is known for its high accuracy, low latency, and efficient training, making it suitable for applications ranging from lightweight deployments to high-performance computing.

Inception-v4 (29) integrates a Stem module for preliminary feature extraction, Inception-A/B/C modules to capture fine-grained and multi-scale semantic features, Reduction-A/B modules for spatial downsampling, and a final classification head. This design enables the effective extraction of hierarchical and multi-scale features for complex image classification tasks.

MobileNetV3 (30) is composed of a Stem module, a series of Bottleneck blocks, and a Squeeze-and-Excitation attention mechanism, and incorporates neural architecture search components. It achieves significant reductions in parameter count and computational complexity without compromising classification accuracy.

ResNet18 (31) comprises a 7 × 7 convolutional layer with a stride of 2, followed by 16 3 × 3 convolutional layers arranged in 8 residual blocks (each with two convolutional layers), and a fully connected output layer. Despite its relatively shallow depth, ResNet18 exhibits strong representational power and is widely used in image classification, object detection, and embedded vision applications.

VGG16 (32) features a straightforward design consisting solely of stacked 3 × 3 convolutional layers and 2 × 2 max-pooling layers. With 16 weight layers in total, VGG16 is widely adopted in computer vision tasks due to its simplicity and transfer learning capability.

To enhance model performance, we fine-tuned each architecture by unfreezing the final block and applying the Softmax function for binary classification. In addition, we added L2 regularization to prevent overfitting. The batch size was set to 32, with a learning rate of 1e-5. Training was performed for up to 50 epochs, and early stopping was implemented to prevent overfitting by terminating training once the optimal model was reached. The preprocessed dataset was randomly partitioned 8:2 into test and training sets. The training and test sets were kept strictly independent to ensure complete separation and prevent any risk of data leakage.

### 3.5 Model evaluation

To assess model performance, we employed several evaluation metrics, including accuracy, precision, recall, F1 score, loss function values, Receiver Operating Characteristic (ROC) curves, and PR curves.

In our binary classification setting, positive and negative labels correspond to PD and healthy samples, respectively. Classification outcomes were categorized as follows: True Positive (TP): PD samples correctly classified as PD; False Negative (FN): PD samples misclassified as healthy; False Positive (FP): Healthy samples misclassified as PD; True Negative (TN): Healthy samples correctly classified. Based on the classifier's predicted probabilities, samples were ranked, and the following metrics were computed.

#### 3.5.1 Accuracy, precision, recall, and F1 score

Accuracy is defined as the ratio of the number of correctly predicted samples to the total number of samples:

$$\mathrm{Accuracy}\;=\frac{\mathrm{TP}+\mathrm{TN}}{\mathrm{TP}+\mathrm{FP}+\mathrm{TN}+\mathrm{FN}}$$

Precision measures the proportion of actual PD samples among those predicted as PD:

$$\mathrm{Precision}\;=\frac{\mathrm{TP}}{\mathrm{TP}+\mathrm{FP}}$$

Recall (Sensitivity) represents the proportion of actual PD samples that are accurately identified:

$$\mathrm{Recall}\;=\frac{\mathrm{TP}}{\mathrm{TP}+\mathrm{FN}}$$

F1 Score is the harmonic mean of precision and recall, reflecting the balance between them:

$$F1\;\mathrm{score}\;=\frac{2\mathrm{TP}}{2\mathrm{TP}+\mathrm{FP}+\mathrm{FN}}$$

#### 3.5.2 Loss function

The loss function quantifies the deviation between the predicted values and the true values, guiding model optimization. In this study, we applied the cross-entropy loss in conjunction with the Softmax activation function. A lower loss indicates better model generalization and predictive accuracy.

#### 3.5.3 Receiver operating characteristic (ROC) curve and precision–recall (PR) curve

The ROC curve is a graphical representation to assess the performance of classification models. It depicts the relationship between the False Positive Rate (FPR) on the *x*-axis and the True Positive Rate (TPR) on the *y*-axis. Area Under Curve (AUC) quantifies the model's overall discriminative ability, with values spanning from 0.5 (indicative of random chance) to 1.0 (indicating perfect classification (33). By comparing the shapes of ROC curves and their corresponding AUC values across models, one can intuitively assess classifier performance. Higher AUC means better differentiation.

FPR=FP/(FP+TN)

TPR=TP/(TP+FN)

The PR curve depicts recall on the *x*-axis and precision on the *y*-axis and is constructed by computing these metrics at various classification thresholds (34). The PR curve is particularly informative in scenarios with class imbalance, as it focuses on the performance concerning the positive class. A curve that approaches the top-right corner of the chart denotes stronger performance. Similarly, PR-AUC provides a summary measure of the balance between precision and recall; a larger PR-AUC reflects a more favorable trade-off and, consequently, better overall model effectiveness.

#### 3.5.4 Comparative statistical testing of models

In the comparative analysis of classification models, we employed a tripartite statistical framework to ensure robust and interpretable results. The McNemar test (35) served as the primary method for evaluating significant differences in classification accuracy between paired models. This non-parametric test examines discordant predictions through a contingency table framework, offering heightened sensitivity to error rate disparities in binary classification tasks compared to traditional chi-square tests, particularly at moderate sample sizes (*n* > 30). Complementing this, paired *t*-tests (36) were applied to detect systematic biases in predicted probability distributions. To address potential non-normality in probability outputs, we concurrently implemented the Wilcoxon signed-rank test (37) for capturing skewness, heavy tails, or outlier-driven distributional asymmetries. This methodological triangulation creates a synergistic analytical framework: the McNemar test directly quantifies classification efficacy differences, paired *t*-tests reveal central tendency biases, and Wilcoxon tests characterize distributional divergence.

#### 3.5.5 Model interpretability

To enhance the interpretability of our models, we employed Gradient-weighted Class Activation Mapping (Grad-CAM) (38), visualizing the region in the input image that contributes the most to model decision-making. By generating activation heatmaps overlaid on facial images, Grad-CAM enables insight into which facial areas influenced the classification outcome. We implemented Grad-CAM on the final convolutional layer of each model to localize discriminative regions relevant to PD identification.

## 4 Results

### 4.1 Participants

To minimize variability and ensure sample accuracy, we excluded images in which participants were blinking or displaying expressions influenced by nervousness. From the recruited cohort, we selected 200 patients whose images captured a natural, neutral facial expression. To achieve balanced group distribution and maintain statistical robustness, 1,000 individuals were randomly selected for the control group. The patient group comprised 114 males and 86 females, with a mean age of 65.83 ± 9.74 years, while the control group included 572 males and 428 females, with a mean age of 64.95 ± 10.24 years.

### 4.2 Data preparation and model training

Following data cleaning, we selected 1,000 eligible facial images each from the enrolled patients and the recruited healthy individuals. After data augmentation, the number of images in each group increased to 3,000. The two datasets were separated into training and test sets randomly (mutually independent) at a ratio of 8:2, and fine-tuned training was conducted on the models of EfficientNetV2, Inception-v4, MobileNetV3, ResNet18, and VGG16 respectively.

### 4.3 Performance parameters

The classification performances including Accuracy, Precision, Recall (Sensitivity), Specificity, and F1 Score of the five models are listed in Table 2. As evident from the tabulated results, the models exhibit varying levels of effectiveness across the evaluation metrics.

**Table 2:** Accuracy, precision, recall (sensitivity), specification, and F1 score for each model.

Model	Accuracy (%)	Precision (%)	Recall (sensitivity) (%)	Specificity (%)	F1 score (%)
EfficientNetV2	96.33	97.12	95.50	97.17	96.30
Inception-v4	87.42	88.38	86.17	88.67	87.26
MobileNetV3	98.83	98.67	99.00	98.67	98.84
ResNet18	99.67	99.50	99.83	99.50	99.67
VGG16	98.42	98.18	98.67	98.17	98.42

Among them, ResNet18 demonstrates the most outstanding overall performance, with its accuracy, recall, specificity, and F1 score all approaching or reaching 99.67%, marginally outperforming others. Next are MobileNetV3 and VGG16. MobileNetV3 shows a high accuracy rate, while VGG16 exhibits a well-balanced classification ability. In particular, MobileNetV3 achieves a recall rate of 99.00%. EfficientNetV2 also performs stably, with an F1 score of 96.30%, which is superior to that of Inception-v4. In contrast, Inception-v4 has relatively lower values in all metrics, with an F1 score of 87.26%, indicating that its recognition ability in this task is slightly inferior. In summary, considering their performance in this specific dataset, ResNet18 and MobileNetV3 emerge as the most effective models and are recommended as the preferred architectures for this application scenario.

### 4.4 Accuracy curves

**Figure 2:**
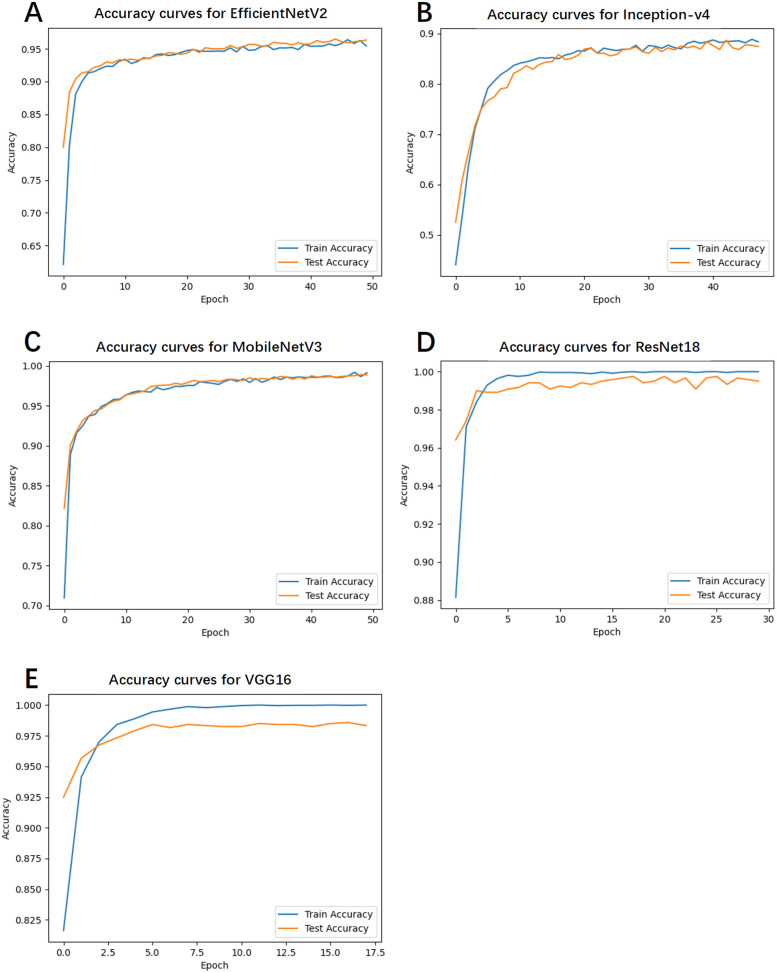
The accuracy curves for both training and test sets across all models during training. **(A)** Accuracy curves for EfficientNetV2. **(B)** Accuracy curves for Inception-v4. **(C)** Accuracy curves for MobileNetV3. **(D)** Accuracy curves for ResNet18. **(E)** Accuracy curves for VGG16.

Figure 2 illustrates the accuracy curves for both training and test sets across all models during training. With respect to convergence speed, VGG16 and ResNet18 converged most rapidly, followed sequentially by Inception-v4, MobileNetV3, and EfficientNetV2. Due to the implementation of an early stopping mechanism, the first three models terminated training prematurely upon reaching optimal and stabilized performance.

### 4.5 Training and test loss

**Figure 3:**
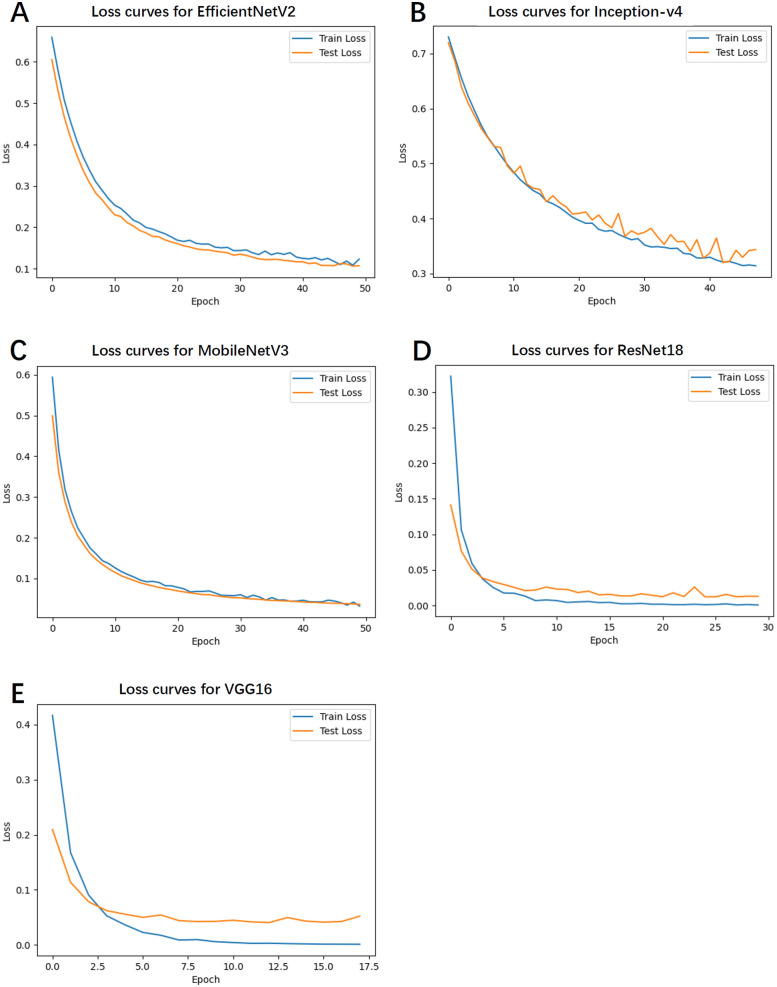
The loss curves for the training and test sets. **(A)** Loss curves for EfficientNetV2. **(B)** Loss curves for Inception-v4. **(C)** Loss curves for MobileNetV3. **(D)** Loss curves for ResNet18. **(E)** Loss curves for VGG16.

Figure 3 depicts the loss curves for the training and test sets. Notably, EfficientNetV2 and MobileNetV3 exhibited smooth and steadily declining loss values, indicating high training stability and a lower likelihood of overfitting. Conversely, Inception-v4 and VGG16 demonstrated fluctuations in the latter stages of training, with slight increases in loss, suggesting a potential overfitting risk.

### 4.6 ROC and PR curve analysis

**Figure 4:**
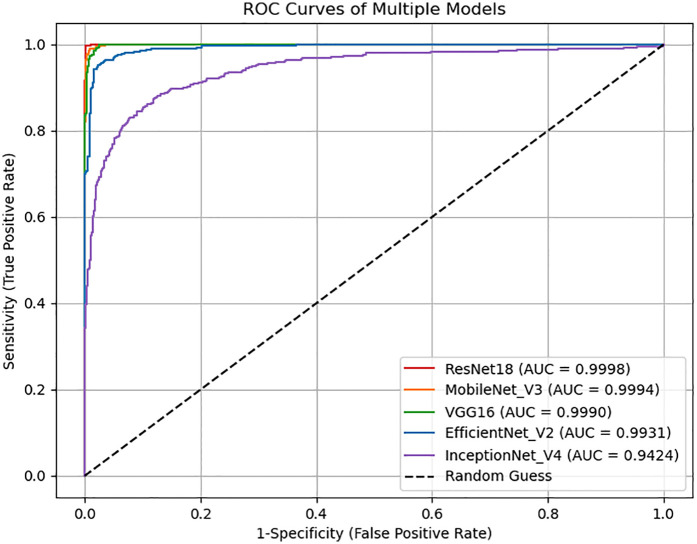
The ROC curve for each model.

**Figure 5:**
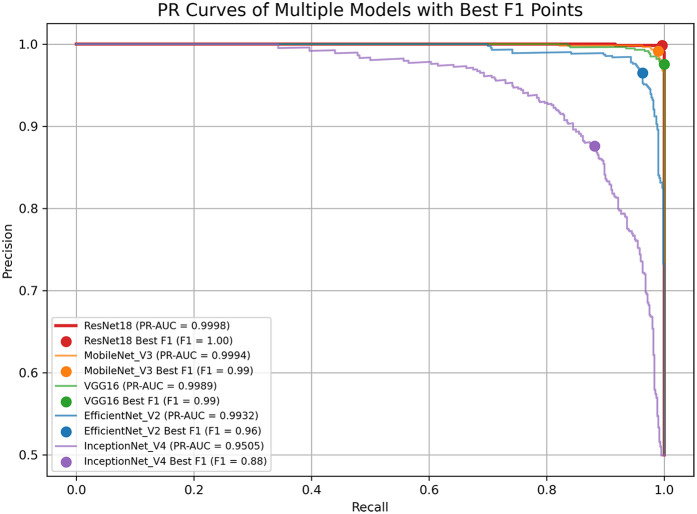
The PR curve for each model.

Figures 4, 5 display the ROC and PR curves, respectively. The PR curve reflects the balance between precision and recall across various classification thresholds, whereas the ROC is the relationship graph between TPR and FPR, serving as a comprehensive metric of overall classification performance. Among the models, ResNet18, MobileNetV3, and VGG16 delivered superior performance, as indicated by their higher AUCs. In contrast, Inception-v4 underperformed, which may be attributed to model overfitting or suboptimal parameter settings.

### 4.7 Comparative statistical analysis of model performance

The pairwise statistical comparisons of model performance are summarized in Table 3. ResNet18 emerged as the top-performing model, demonstrating a clear and consistent advantage across all key metrics. Its accuracy exceeded that of EfficientNetV2 by 3.33% (*p* = 4.12 × 10^−^⁹), Inception-v4 by 12.25% (*p* = 5.70 × 10^−^³³), MobileNetV3 by 0.83% (*p* = 0.024), and VGG16 by 1.25% (*p* = 0.0013), with all differences reaching statistical significance. MobileNetV3 (98.83%) and VGG16 (98.42%) formed a high-performance second tier, showing no significant difference between them (*p* = 0.404), but both significantly outperformed the remaining models. EfficientNetV2 achieved moderate performance—significantly better than the lowest-ranked Inception-v4 (accuracy difference of 8.92%, *p* = 2.28 × 10^−^¹⁸)—yet still markedly inferior to the top three models. Inception-v4 consistently ranked last, with an accuracy of 87.42% and an AUC of 94.24%, and exhibited highly significant disadvantages compared with all other models (*p* < 10^−^²⁷). The primary source of performance disparity lay in the ability to identify positive cases (sensitivity), with ResNet18 showing particular superiority in handling borderline or ambiguous samples (e.g., a 42:2 win ratio against EfficientNetV2 in contested cases).

**Table 3:** Comparative statistical analysis results of model performance.

Model 1	Model 2	McNemar *P* value	Paired t-test *P* value	Wilcoxon *P* value	Accuracy difference	F1 difference	Sensitivity difference	Specificity difference	Discord-ant pairs	Model 1 win pairs	Model 2 win pairs
EfficientNetV2	Inception-v4	2.28E-18	0.5,12,361	0.179463	0.089167	0.090451	0.093333	0.085	147	127	20
EfficientNetV2	MobileNetV3	5.78E-05	0.158766	0.523516	–0.025	–0.02533	–0.035	–0.015	52	11	41
EfficientNetV2	ResNet18	4.12E-09	0.143687	0.838453	–0.03333	–0.03365	–0.04333	–0.02333	44	2	42
EfficientNetV2	VGG16	0.000778	0.160508	0.994153	–0.02083	–0.02118	–0.03167	–0.01	51	13	38
Inception-v4	MobileNetV3	1.80E-28	0.888871	0.406785	–0.11417	–0.11578	-0.12833	–0.1	151	7	144
Inception-v4	ResNet18	5.70E-33	0.827447	0.561822	–0.1225	–0.1241	-0.13667	–0.10833	149	1	148
Inception-v4	VGG16	4.87E-27	0.85035	0.570785	–0.11	–0.11163	–0.125	–0.095	148	8	140
MobileNetV3	ResNet18	0.024449	0.77887	0.858788	–0.00833	–0.00832	–0.00833	–0.00833	16	3	13
MobileNetV3	VGG16	0.404248	0.883736	0.917131	0.004167	0.004147	0.003333	0.005	23	14	9
ResNet18	VGG16	0.001319	0.92042	0.000187	0.0125	0.012466	0.011667	0.013333	19	17	2

### 4.8 Model interpretation via grad-CAM

**Figure 6:**
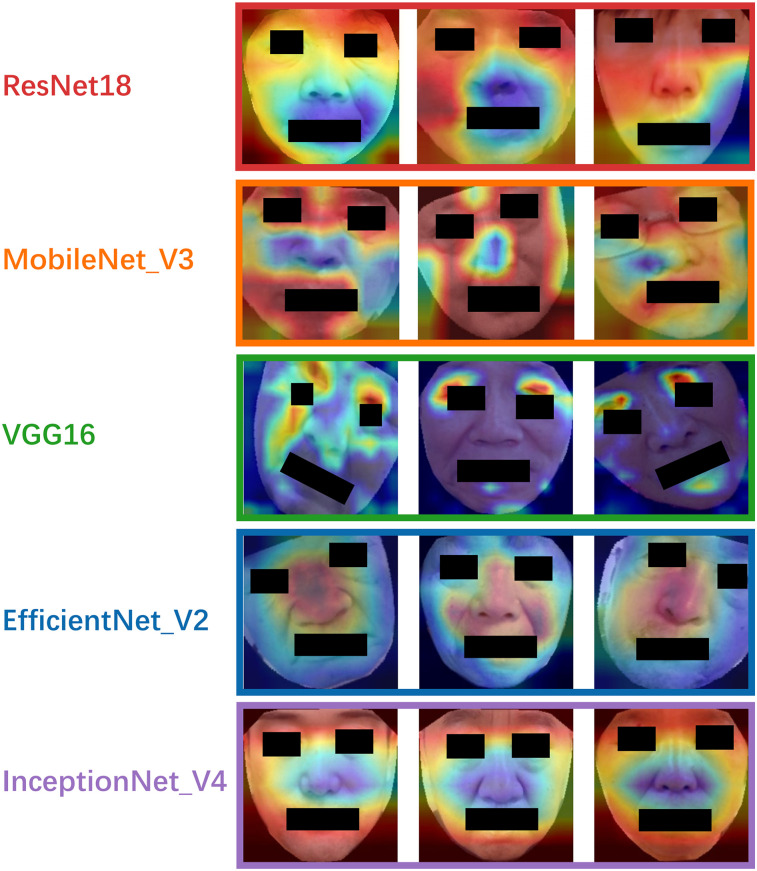
The gradient-weighted class activation mapping for each model.

To enhance model interpretability, Grad-CAM heatmaps were generated for each model, as shown in Figure 6. The heatmaps revealed that ResNet18 primarily focused on the upper facial region above the nose, while MobileNetV3 and Inception-v4 concentrated on the periorbital and perioral areas. VGG16 showed strong attention to the eyes and lips, and EfficientNetV2 concentrated on the nasal region and its surroundings. Although attention varied across models, the eyes and lips emerged as the most consistently emphasized regions, suggesting their potential relevance as discriminative facial features in PD classification.

## 5 Discussion

PD, as a neurodegenerative disorder, has a variety of manifestations in both motor and non-motor symptoms (39). One of its hallmark features is hypomimia, which is characterized by a reduction or absence of facial expressions, resulting in an unnatural facial appearance (40, 41). This characteristic facial feature can serve as a crucial clue in PD diagnosis. In this study, we evaluated five classical CNN models for the identification of early and mid-stage PD based on static facial images. The experimental results demonstrate that CNNs are capable of effectively capturing discriminative facial features associated with PD.

The five models evaluated in this study represent a diverse spectrum of convolutional neural network architectures, ranging from lightweight to high-performance designs. This selection includes the latest efficient architecture (EfficientNetV2), well-established benchmarks widely validated in medical image analysis (ResNet18, VGG16), and multi-branch networks with strong fine-grained feature extraction capabilities (Inception-v4). Compared with other lightweight alternatives, such as EfficientNet Lite and MobileViT, these architectures offer greater training stability and stronger interpretability on small-sample medical datasets, supported by extensive prior literature in similar tasks. Such characteristics facilitate horizontal comparisons and reproducibility. Collectively, this model set enables a comprehensive assessment of architectures with different scales and design philosophies for facial image–based Parkinson's disease recognition, while maintaining high diagnostic accuracy.

Among the models tested, ResNet18 consistently outperformed the others across all evaluation metrics, achieving the highest accuracy, recall, specificity, and F1 score. This superior performance may be attributed to its residual structure, which facilitates gradient flow and mitigates vanishing gradient issues during deep network training (42). MobileNetV3 also showed competitive performance, particularly in the recall, making it a promising lightweight alternative suitable for deployment in mobile or embedded diagnostic platforms. Due to the accompanying aging-related phenomena in the elderly population, many clinical manifestations of PD are highly likely to be overlooked, leading to delays in diagnosis and treatment. In this context, achieving a high recall rate in predictive models is particularly valuable, as it represents the model's capability to precisely recognize genuine positive cases, especially rare ones, thereby minimizing the risk of misdiagnosis. VGG16 exhibited fast convergence during training, reaching optimal performance rapidly under the early stopping mechanism. While its final accuracy and recall were slightly lower than those of ResNet18, it maintained a well-balanced classification ability across all metrics. EfficientNetV2 showed stable training performance with a steadily declining loss curve for both training and test sets, suggesting a strong resistance to overfitting. Its performance metrics, particularly the F1 score (96.30%), indicate robust and balanced classification capabilities. In contrast, Inception-v4 exhibited the weakest performance, as indicated by relatively lower accuracy and F1 score values. The loss curve of Inception-v4 displayed signs of instability during the later stages of training, possibly reflecting sensitivity to parameter tuning or insufficient adaptation to the facial feature distribution in the dataset. These results suggest that not all complex architectures are necessarily advantageous for small-to-moderate-scale facial classification tasks, especially when the features are subtle. The ROC and PR curve analyses further supported these findings. ResNet18 and MobileNetV3 achieved the largest areas under both the ROC and PR curves, reflecting their strong discriminative capabilities and robust trade-offs between precision and recall. Meanwhile, the modest AUC values of Inception-v4 indicate suboptimal classification boundaries and weaker generalization ability. Our best-performing model, ResNet18, achieved the highest recognition accuracy on real facial images (as opposed to synthetically generated images) when compared with models reported in previous studies (Table 1).

To enhance model interpretability, we applied Grad-CAM. Although different models attended to various facial regions, the worth noticing is that eyes and lips emerged as consistently important across multiple models. Studies proved that upper facial dyskinesia in PD patients is most evident in the reduced frequency of spontaneous blinking and prolonged pauses between eyelid closure and reopening (43). Lower facial motor abnormalities are primarily reflected in expressive movements such as smiling. For instance, the peak velocity and amplitude of lip corner movements during postural or voluntary smiles are notably reduced, with these kinematic deficits strongly correlating with the severity of bradykinesia in the limbs (44). Studies employing facial electromyography and action unit analysis have revealed significantly reduced muscle activity in the periocular region and at the corners of the mouth in PD patients compared to healthy controls (45, 46). Our results are consistent with known the regions of PD-related hypomimia, such as reduced blinking, slackened lips, and diminished expressiveness in the mid-facial area. This visual evidence provides physiological plausibility to the models' decisions and may serve as a foundation for identifying explainable facial biomarkers of PD. From a clinical perspective, these findings demonstrate the potential of AI-based facial image analysis for non-invasive, cost-effective, and scalable screening of early and mid-stage PD, particularly in remote or resource-limited settings where traditional diagnostic tools may be inaccessible. Moreover, the models explored here lay the groundwork for further integration into telemedicine platforms and mobile health applications.

Despite the promising results, several limitations should be noted. First, the dataset utilized in our research was collected under relatively controlled conditions, with uniform lighting, background, and head positioning. Consequently, the generalizability of the models to real-world clinical or other settings remains to be tested, such as scenarios with cluttered backgrounds, varying lighting conditions and angles, non-neutral facial expressions, and device disparities. Although data augmentation has been employed to achieve data diversity and balance the cohort size, this does not represent real-world population differences and cannot substitute for genuine population diversity, especially regarding variations caused by gender, age group, race, skin color, facial features, or comorbidities. Future studies will focus on expanding the dataset to include larger and more heterogeneous populations, and validating the models in real-world clinical environments. Moreover, both the model training and testing were conducted using data from the same source and device, lacking multi-center data testing. In the future, a larger-scale and more diverse dataset from different sources and collection methods is required to enhance the robustness of the models in practical applications. This study focused exclusively on static facial images, which inherently omits dynamic facial cues such as blinking frequency, micro-expressions, and subtle movement patterns. Although this choice was intentional to simplify data acquisition, reduce computational cost, and improve applicability in low-resource or remote settings, it may limit the system's ability to capture certain early Parkinsonian signs associated with facial dynamics. Future work could incorporate short video segments or frame sequences to extract temporal features via 3D CNNs or transformer-based models, potentially enhancing sensitivity to subtle motor impairments while maintaining usability in clinical and telehealth environments. The interpretation of Grad-CAM is still an empirical inference, and the regions of interest in the model may be interfered with by facial textures or lighting during image capture. Further efforts towards enhancing model interpretability and clinical explainability are crucial to foster trust and adoption among healthcare professionals. The facial-image–based screening classifier for early- to mid-stage Parkinson's disease should be positioned as a highly sensitive, auxiliary tool to support screening and clinical decision-making. When integrated into existing clinical workflows, it can facilitate risk stratification, generate automated referral recommendations, and support the primary care, telemedicine, and specialist settings, thereby improving early presentation rates and enhancing diagnostic efficiency. Before clinical deployment, the system should undergo rigorous multi-center external validation and prospective pilot studies to assess its performance and fairness across diverse populations. It must also comply with relevant regulatory requirements, ensuring robust privacy protection and data security. From a technical perspective, integration with hospital Hospital Information System via Fast Healthcare Interoperability Resources standards is recommended, alongside the provision of interpretable outputs (e.g., Grad-CAM heatmaps and associated confidence scores). A comprehensive quality management framework and post-deployment performance monitoring should be established to mitigate potential risks, such as false positives or model drift. Furthermore, embedding the most effective models into portable or smartphone-based platforms could enable large-scale, low-cost screening and facilitate ongoing external validation, particularly in underserved or remote regions. Given its non-invasive, scalable, and economically viable nature, this approach offers significant clinical and societal benefits for early detection of Parkinson's disease in aging populations.

## 6 Conclusion

This study demonstrates the feasibility and effectiveness of using CNNs to classify early to mid-stage PD based on static facial images. Among the five architectures evaluated, ResNet18 and MobileNetV3 emerged as the top-performing models, offering both high accuracy and interpretability through Grad-CAM analysis. These findings suggest that facial phenotypes—particularly features related to ocular and oral muscle activity—can serve as viable, non-invasive biomarkers for early PD screening. The proposed approach holds significant promise for deployment in scalable, low-cost diagnostic applications, potentially contributing to earlier intervention and improved patient outcomes.

## Data Availability

The datasets presented in this article are not readily available due to the privacy concerns of the original data, namely facial images, we have signed a confidentiality agreement with the subjects and therefore cannot disclose it. Requests to access the datasets should be directed to Wei Yan, sdweirui2018@163.com.
